# Food Systems Transformation for Child Health and Well-Being: The Essential Role of Dairy

**DOI:** 10.3390/ijerph181910535

**Published:** 2021-10-08

**Authors:** Gregory D. Miller, Mitch Kanter, Laurence Rycken, Kevin B. Comerford, Nicholas M. Gardner, Katie A. Brown

**Affiliations:** 1 National Dairy Council, Rosemont, IL 60018-5616, USA; gregory.miller@dairy.org (G.D.M.); katie.brown@dairy.org (K.A.B.); 2 Global Dairy Platform, Rosemont, IL 60018-5616, USA; Mitch.Kanter@GlobalDairyPlatform.com; 3 International Dairy Federation, 1030 Brussels, Belgium; lrycken@fil-idf.org; 4 California Dairy Research Foundation, Davis, CA 95618, USA; 5 U.S. Dairy Export Council, Arlington, VA 22201, USA; ngardner@usdec.org

**Keywords:** child health, child nutrition, dairy, food security, food systems, nutrition, sustainability, sustainable food systems

## Abstract

Malnutrition, in all its forms, during the critical stages of child growth and development can have lifelong impacts on health and well-being. While most forms of malnutrition can be prevented with simple dietary interventions, both undernutrition and overnutrition remain persistent and burdensome challenges for large portions of the global population, especially for young children who are dependent on others for nourishment. In addition to dietary factors, children’s health also faces the growing challenges of climate change, environmental degradation, pollution, and infectious disease. Food production and consumption practices both sit at the nexus of these issues, and both must be significantly transformed if we are to achieve the 2030 Sustainable Development Goals. Food sources (i.e., animal-source foods vs. plant-source foods), food production practices, the effects of food processing, the impacts of a more globalized food system, and food loss and waste have all been receiving growing attention in health and sustainability research and policy discussions. Much of this work points to recommendations to reduce resource-intensive animal-source foods, heavily processed foods, and foods associated with excessive waste and pollution, while simultaneously increasing plant-source options. However, some of these recommendations require a little more nuance when considered in the context of issues such as global child health. All types of foods can play significant roles in providing essential nutrition for children across the globe, and for improving the well-being and livelihoods of their families and communities. Dairy foods provide a prime example of this need for nuance, as both dairy production practices and consumption patterns vary greatly throughout the world, as do their impacts on child health and food system sustainability. The objective of this narrative review is to highlight the role of dairy in supporting child health in the context of food system sustainability. When considering child health within this context it is recommended to take a holistic approach that considers all four domains of sustainability (health, economics, society, and the environment) to better weigh trade-offs, optimize outcomes, and avoid unintended consequences. To ensure that children have access to nutritious and safe foods within sustainable food systems, special consideration of their needs must be included within the broader food systems transformation narrative.

## 1. Introduction

Supporting the health and well-being of children today is foundational to building a successful future for generations to come. Proper nourishment throughout childhood and adolescence is critical to supporting growth, development, and learning, as well as productivity and overall health later in life [1,2]. Despite the abundance of scientific evidence and global efforts to improve early life eating patterns, malnutrition, in all its forms, remains one of the most significant challenges to child health around the world. Global rates of undernutrition and overweight and obesity continue to rise, and one out of three children under the age of 5 is failing to thrive due to poor dietary patterns [3]. The global population is projected to grow from 7.7 billion today to nearly 10 billion by 2050, with most of this growth likely to occur in parts of the world where malnutrition is highest and healthy diets are most unaffordable [4]. Without proper nutritional interventions, the future of child health will continue to worsen, resulting in future generations being less healthy than current ones. The problem of growing global rates of childhood malnutrition does not arise in isolation, nor can it be resolved through an approach that only focuses on improving dietary patterns. Childhood malnutrition is inextricably linked to several other global sustainability issues; and while inadequate food intake and poor diet quality are both leading causes of childhood malnutrition, these are just symptoms of deeper and more systemic issues such as poverty and food insecurity. At present, food insecurity affects roughly one-quarter of the global population and has been consistently increasing at the global level for years, affecting the diets and health status of hundreds of millions of children around the world [5].

In addition to malnutrition, poverty, and food insecurity, the present generation of children must also contend with global health issues such as climate change, water scarcity, pollution, and global pandemics. These issues all affect, and are all affected by, food systems and dietary patterns. For example, COVID-19 has worsened all forms of malnutrition across the globe, causing significantly more wasting and stunting in the world’s children [6]. This pandemic has also forced more than 1.6 billion children out of school around the world, putting critical school meals out of reach for many who rely on them for consistent daily nourishment [7]. While the COVID-19 pandemic will eventually become less of a threat to children’s well-being, climate change will likely be more of a threat, and the devastation from both underscore the imminent need for all food system actors to prepare for future global crises by transitioning food systems to be more resilient to future shocks.

In the face of climate change and COVID-19, a ‘business-as-usual’ approach to food systems will be disastrous for the planet and its populations [8,9]. Multiple aspects of food systems need to drastically change if we are to achieve the 2030 Sustainable Development Goals (SDGs). A growing body of literature on healthy and sustainable diets points to recommendations to reduce resource-intensive animal-source foods, heavily processed foods, and foods associated with excessive waste and pollution, while simultaneously increasing plant-source options ([10,11,12,13,14]). However, some of these recommendations require a little more nuance when considered in the context of issues such as global child nutrition and health. For example, there are many plant-source and animal-source foods that are resource intensive, heavily processed, rich in nutrients to limit (i.e., salt, sugar, unhealthy fats), associated with high levels of food loss or waste, and/or linked to inordinate pollution and greenhouse gas (GHG) emissions. Yet, there are also many plant-source and animal-source food options that are minimally processed, locally available, relatively low in associated sustainability impacts, and rich in the exact nutrients that children need for proper growth, development, and immune function. For both health and sustainability purposes, the latter options should be prioritized in food systems transitions, regardless of whether they are sourced from plants or animals.

Among animal-source foods, meat and dairy are often singled out as food groups to limit in the sustainable food systems literature. However, these food groups each contain a wide variety of dietary options that are both nutritionally and sustainably heterogenous [15]. In other words, these food groups are not equivalent to each other, nor are they uniform in their makeup. Due to their nutritional and health distinctions, meat and dairy are treated very differently in global food-based dietary guidelines (FBDGs), with dairy being more commonly recommended than meat (dairy is recommended in >70% of FBDGs, while meat in recommended in <55%), and also recommended in higher frequency (dairy is recommended an average of one to four servings/day in FBDGs, meat is recommended at approximately five servings per week) [16,17]. Regarding sustainability, the differences between environmental impacts such as carbon footprint can range up to 25-fold between dairy and meat products, with intensive milk production systems being associated with some of the lowest emissions intensities among animal-source foods [15]. The global dairy sector is also linked to nearly 1 billion livelihoods, which is significantly more than any other food sector [18], and leaders across the sector are currently financing a growing number of science-based efforts toward more sustainable food systems [19,20,21,22]. Taken together, these factors make dairy an intriguing case study for its role in the health and sustainability of food systems for present and future generations. Therefore, the objective of this review is to highlight the roles of dairy in supporting child health and well-being in the context of food systems transformation.

## 2. The Global State of Child Nutrition

The present state of our global food systems and dietary patterns are failing to help millions of children meet their nutritional needs. Globally, 45% of deaths in children under the age of 5 are associated with undernutrition [23]. 144 million children among this age group are stunted, 47 million suffer from wasting, and 38 million are overweight [24]. While the prevalence of stunting has been declining globally since 2000, its prevalence is still increasing in regions such as Africa. At present, wasting is highest in Asia and Oceania, where 1 in 10 children under the age of 5 has an increased risk of death due to wasting [24]. In all regions, the rising prevalence of overweight children under the age of 5 is concerning. Most of these issues are predicted to worsen due to the dual crises of COVID-19 and climate change [25].

Inadequate food intake and poor diet quality (imbalanced nutrient intake and/or imbalanced energy intake) are two of the major drivers of malnutrition. Globally, about half (53.1%) of children aged 6–23 months reach the minimum recommended frequency of meals, with only 29.3% meeting minimum dietary diversity and 18.9% meeting the minimums for an acceptable diet for healthy growth and development [26]. Across childhood and adolescence, dietary patterns tend to be lower than national recommendations in nutrient-rich foods including fruits, vegetables, animal-source foods (e.g., meat, dairy, poultry, eggs, and fish) and fortified foods [26]. In all geographic regions, inadequate fruit and vegetable intake is common. Among adolescent girls in low- and middle-income countries (LMICs), only 16% meet the daily serving recommendation for dairy and 46% meet dietary recommendations for meat, poultry, or fish [27]. Diets with low intakes of fruits, vegetables, and animal-source foods put children at risk for nutrition deficiencies, with many of the micronutrients that are missing in children’s diets globally (iron, calcium, zinc, folate, vitamin B12, vitamin D) found mainly in animal-source foods [28].

Not only are poor diets the leading cause of many forms of childhood undernutrition; they are also a leading cause of childhood overweight and obesity. Globally, more than 38 million children under the age of 5 years are overweight or obese, and more than 340 million between the ages of 5–19 years are overweight or obese [29]. Once considered only an issue in high-income countries (HICs), overweight and obesity are increasing in LMICs, which now must face the double-burden of malnutrition, encompassing the impacts of undernutrition as well as obesity and overweight. For example, since 2000, the number of overweight children under the age of 5 in Africa has risen by nearly 24%, while in Asia, almost half of the children under the age of 5 are overweight or obese according to the World Health Organization (WHO) [29]. Unfortunately, large data gaps exist on overall dietary quality in children, limiting characterization of the effects of early life dietary quality over the course of life [30].

Childhood overweight and obesity are major risk factors for non-communicable disease (NCD)-related deaths from cardiovascular disease (CVD), type 2 diabetes (T2D), cancer, and musculoskeletal disorders (e.g., osteoarthritis, osteoporosis) [1,31]. These largely preventable diseases claim more than 40 million lives each year, or nearly three-quarters of all global deaths [32]. The pathophysiology and related symptoms of many diet- related NCDs begin in childhood, creating a variety of costs and offsets that often last for decades, including detriments to physical health, social and economic status, and overall quality of life. These costs and offsets also carry over to social institutions, affecting the time and resources of families, schools, work, government programs, and healthcare systems. It is estimated that the global cost of CVD alone will rise from approximately $863 billion in 2010 to over $1 trillion by 2030, further underscoring the value of focusing on child nutrition and healthy weight early in life [33]. Prioritizing the nutrition and health of the young, especially those who are poor and/or vulnerable, is not only a way to prevent unnecessary suffering and social costs, but also a way towards improving the health and well-being of all future populations as well as the economic stability of our future social systems [34].

In addition to the negative effects on physical health (i.e., body size, body weight, risk for NCDs), poor childhood nutrition is also associated with substandard neurological development, cognitive function, and psychosocial outcomes [2,35]. These issues manifest in various ways, such as impairments to visual processing, attention, memory, motor skills, and overall intelligence. While many of these early impairments can have lifelong effects [36], many others can be minimized or reversed with early life dietary improvements [2]. Taken together, these factors highlight the massive impacts that early childhood nutrition can have on physical and mental health and show that a global shift towards healthier diets could greatly reduce our population’s current and future disease burden.

## 3. Global Efforts Pushing Towards Healthier Diets for Children and More Sustainable Food Systems

In 2016, The United Nations (UN) established a set of 17 SDGs to “promote prosperity while protecting the planet” [37]. SDG #2, “Zero Hunger” aims to end hunger, achieve food security, improve nutrition, and promote sustainable agriculture by 2030,unifying healthy diets and sustainable food systems in one important goal. However, at present, progress in the fight against global malnutrition is insufficient to meet SDG targets for 2030 [24], with the prevalence of multiple forms of malnutrition among children under the age of 5 remaining stubbornly high year-over-year [5]. Alongside persistent malnutrition, children face the additional challenges of current and future impacts of environmental degradation from their food systems and consumption patterns. And while climate change receives the bulk of attention, there are many more sustainability issues that rarely receive media headlines.

Therefore, the UN and its collection of specialized organizations that prioritize child health and sustainability (e.g., WHO, United Nations Children’s Fund (UNICEF), and the Food and Agriculture Organization (FAO)), are making it clear that sustainable food systems are about more than protecting the environment and improving dietary patterns. There are also numerous social and economic factors to consider for current and future generations. For example, the UN’s Committee on World Food Security High Level Panel of Experts on Food Security and Nutrition (CFS HLPE) defines sustainable food systems as the intention “to provide food security and nutrition today, in such a way that does not compromise the environmental, economic, and social bases that generate food security and nutrition for future generations” [38]. The recent UN Food Systems Summit, which took place in September of 2021, echoed this sentiment. The UN Secretary-General made it clear that in addition to nourishing people and respecting nature, sustainable and resilient foods systems must also emphasize equitable livelihoods, decent work and empowered communities [39]. Summit participants are aiming to put these recommendations into action by supporting two new multi-stakeholder and multi-sectoral coalitions, one with a focus on healthy diets from sustainable food systems and the other focused on providing healthy school meals to children [40]. Also, with an eye towards the future, leaders of the 2021 Food Systems Summit convened a Global Youth Summit Dialogue focused on the critical roles of youth in achieving the SDGs and laying the groundwork for sustainable food systems transformation [41]. This call to action to food system actors to be more inclusive of younger generations is also reflected in shifting policy recommendations, such as in global FBDGs that are placing more focus on childhood health [42], and a more holistic approach to dietary patterns that are inclusive of sustainability factors [43].

## 4. Factors Affecting Food Systems Transformation

There are numerous ways to assess the health and sustainability impacts of foods, with factors such as food source (i.e., animal-source foods vs. plant-source foods), protein quantity and quality, and carbon footprint receiving significant attention of late [11,44,45,46,47]. Although these are all critical issues for achieving healthier diets from more sustainable foods systems, they by no means comprise a comprehensive list. There are hundreds of compounds in foods that matter for health (e.g., vitamins, minerals, amino acids, fatty acids, fibers, probiotics, phenolics), hundreds of factors that matter for food system sustainability (e.g., carbon footprint, water footprint, land use, fertilizer use, cost of production, food lost or wasted, eutrophication, food accessibility and affordability) and many ways to measure and compare foods (per kg, per 100 kcal, per serving, per g of nutrient, etc.). Any combination of these variables may provide useful information for understanding the health and sustainability impacts of foods or diets.

Furthermore, these variables are constantly changing, making it difficult to predict how altering one aspect (e.g., pesticide us, fertilizer use, grazing schedule) may affect another (e.g., biodiversity, crop pollination, crop yield). While certain indicators may be more important than others for global health and sustainability, decision-making that only focuses on simple or singular indicators (e.g., carbon footprint/kg food) will likely lead to missed opportunities to mitigate tradeoffs or capitalize on food system synergies. For example, issues such as intensification, incentivization, and subsidization are potential game changers when it comes to improving the health and sustainability of food systems, but at the same time, these types of policies and practices can also have unintended consequences across all sustainability domains [48]. Similarly, research models show that reducing a major food system variable, such as the availability of animal-source foods in the food supply, may moderately reduce GHG emissions, but at the same time negatively impact hundreds of millions of agricultural livelihoods, as well as the availability of essential nutrients in the food supply, and the overall well-being of a large portion of the global population [49,50,51]. If we are to collectively achieve the SDGs by 2030, all major food system actors must unite behind “win-win” (i.e., synergistic) solutions that aim to solve multiple sustainability issues at once without causing new ones [52]. Accomplishing this task of optimizing food systems transformation will require a huge shift in industry and government support towards transdisciplinary approaches to research and policy decisions, with many of these new strategies dependent on the development and deployment of evidence-based innovations and technologies [53,54]. These “win-win” science-backed strategies, such as food fortification, regenerative agriculture, sustainable intensification, carbon capture/sequestration, blockchain technologies, upcycling of byproducts, waste valorization, improvements in biofuels, and the creation of safer agrochemicals [55,56,57,58], will all play key roles towards enabling safer, healthier, and more sustainable futures for the next generations.

## 5. Dairy’s Essential Role in Child Health Within Sustainable Food Systems

### 5.1. Meeting the Nutrition and Health Needs of Children

To meet the nutrient needs necessary for proper growth and development, children require a balanced and varied diet of safe, nutrient-rich foods. In general, FBDGs from around the world recommend that these nutrient-rich foods come from both plant- and animal-source food groups on a daily basis [17]. The most commonly recommended food groups in FBDGs are fruits/vegetables and dairy foods-with approximately 70% of FBDGs recommending daily intake of milk and other dairy foods, and most of the other 30% of FBDGs consolidating dairy food recommendations into those for “protein foods” or “foods from animals” [17]. Among animal-source food groups, dairy foods, such as milk, cheese, and yogurt, contain an unmatched set of essential nutrients, which are rich in several of the nutrients of public health concern for underconsumption in both LMICs and HICs (e.g., calcium, iodine, potassium, zinc, vitamin A, vitamin D) [42,59]. Dairy foods are primarily recommended in FBDGs because they contribute nutrients critical for healthy growth and development, including calcium, vitamin D and high-quality protein [16]. Consuming adequate amounts of these nutrients is especially important to support skeletal health in childhood and adolescence [60]; and emerging research also suggests that the beneficial microbes found in fermented dairy foods may act in concert with calcium and vitamin D to benefit bone health later in life [61].

Although not often mentioned in FBDGs, dairy foods are also rich in magnesium, phosphorus, riboflavin, selenium, and vitamin B12 [42]. Many of the essential nutrients (and bioactive compounds in the case of probiotics) that dairy foods provide are under-consumed globally, and their adequate consumption has been shown to help improve growth in young children and to contribute to proper brain development and/or overall health in humans of all ages [3,42,62,63]. Additionally, the consumption of milk and other animal-source foods by undernourished children in LMICs has been linked to reduced risk of morbidity and mortality as well as improved cognitive function [64]. Obtaining similar quantity and quality of protein and micronutrients strictly from plant-source foods can be challenging for young children, given their small stomach size and the amount of plant-source foods required to meet requirements [65]. Research from the U.S. also shows that replacing dairy foods with nutrient-equivalent non-dairy options can lead to increases in both the cost and energy content of dietary patterns, primarily having to do with the difficulty in achieving nutrient adequacy for calcium and vitamin D from low/no dairy diets [66].

Higher consumption of foods that are sources of vitamin A, B, complex vitamins, vitamin C, vitamin D, zinc, iron, and/or selenium are recommended both for children and various vulnerable populations, including those who are malnourished, immune-compromised, and/or at a higher risk for contracting COVID-19 and other infections [32,67,68]. In the absence of dairy and other animal-source foods in the diet, it can be extremely difficult for young children to meet requirements for several of these micronutrients, unless they are consuming highly fortified options [65]. While fortified foods can provide a safe and cost-effective public health strategy for preventing nutrient deficiencies around the world, several of the main vehicles for fortification tend to be highly processed plant-based ingredients (salt, sugar, flour, oils) [69] which are commonly listed in global FBDGs as dietary components to limit [17]. Although fortified plant-source foods can possess a host of the essential nutrients required to prevent deficiencies, experts regard animal-source foods as the most effective first-line choice in the treatment of mild and moderately undernourished children [70]. The importance of animal-source foods is also recognized by Codex Alimentarius (which is an internationally recognized collection of guidelines and codes aimed at ensuring the safety, quality, and fairness of the international food trade), which requires that ready-to-eat therapeutic foods contain 50% dairy protein, which further demonstrates the importance of high-quality macronutrients for interventions within extremely vulnerable populations [71].

FBDGs inform national governments on food policy, with many of their dairy food recommendations resulting in the inclusion of daily dairy foods in school meals programs across the globe. In the U.S., dairy has been a mainstay in dietary guidelines since their inception, resulting in milk being a staple in U.S. school meal programs and serving as the main food source of nine essential nutrients (i.e., protein, calcium, phosphorus, magnesium, potassium, vitamins A, B12, D and riboflavin) in the diets of U.S. children and adolescents [72]. Healthy school meals that include dairy have been shown to close nutrient gaps and support improved academic performance and behavior [73]. And while not generally considered in FBDGs, many chronic diseases are diet-dependent and frequently begin in childhood, affecting quality of life and well-being throughout adolescence and adulthood. Adequate dairy intake in youth can beneficially impact health later in life [74], and adults who consume higher levels of dairy food, especially fermented dairy foods, may have a lower risk of several chronic diseases such as CVD, T2D, hypertension, and stroke [75].

The scientific evidence clearly shows that the inclusion of adequate dairy foods in children’s diets can benefit their development and overall health in multiple ways (e.g., by preventing nutrient deficiencies, reducing the risk for later-onset chronic disease, providing beneficial probiotics through cultured products, for use as a vehicle for fortification, and as a base ingredient for therapeutic foods) [60,61,62,71,76,77]. The potential health benefits of consuming adequate dairy are well-documented, as are the reductions in healthcare costs and unnecessary suffering from deficiencies, infections, fractures, and NCDs [78,79,80]. In addition to these essential and potential benefits for human nutrition and health, the FAO and WHO also include milk and dairy products among the foods that fit within sustainable healthy diets for several other reasons as well (e.g., acceptability, accessibility, adaptability, and affordability), all of which may can impact child well-being [81].

### 5.2. Helping Families, Communities, and Economies Thrive

Approximately one billion people globally rely on the dairy sector to support their livelihoods and sustain their local communities [18]. Beyond the farm, milk collection, processing, distribution, and retail all generate direct and indirect employment and income. A 2018 systematic literature review on the dairy sector’s impact on poverty reduction demonstrated dairy cow ownership and/or improvement of dairy cow production had a positive impact across several indicators, including gross household income, household nutrition, crop yields, and household expenditure [82]. This finding and its consistency across study types, countries, and indicators demonstrates that engagement in dairy production is a key factor in improved household welfare around the world. The review also provided strong evidence that dairy production can be part of the solution for improving sustainable development, as it can significantly contribute to poverty reduction at both the family and community levels, both of which are directly related to improving child health and well-being outcomes.

Dairy farms and businesses also provide substantial support to the economy in developed countries. In the U.S., dairy farming and production supports more than three million jobs that generate more than $150 billion in annual wages and over $600 billion in overall economic impact [83]. Dairy farmers also strengthen rural economies in the U.S. by promoting economic opportunity, maintaining and modernizing infrastructure, and establishing human services for their communities. For example, the National Milk Producers Federation has provided high-speed broadband internet and connectivity to rural areas through policy advocacy, funding and coordination, and enhanced mapping of coverage to encourage adequate development [84].

Dairy farming also empowers rural women around the world by increasing their income and influence over vocational and household expenditures. Globally, 37 million dairy farms are led by women, and 80 million women are engaged in dairy farming to some extent [85]. Income generated by dairy farming often enables families in LMICs to provide better educational opportunities to their children. For example, a 2006 study which evaluated the impact of a dairy cattle transfer program on Tanzanian families, showed that participating families that were considered highly income insecure prior to the program were able to send their children to secondary school and, in some cases, to more costly private schools, within three to four years of starting the program [82].

For rural, low-income families, household milk production is associated with positive community impacts. A 2016 study examining the impact of livestock distribution by Heifer International in Zambia showed that distribution of dairy cows to a subset of families led to a statistically significant increase in milk consumption among the community [86]. Importantly, even the households that did not receive an animal in the study demonstrated increased milk consumption due to the increase in the availability and affordability of milk within the community. This type of community “spill-over effect” from increased milk production does not only apply to the milk either, as the addition of dairy cows to certain low-income communities can also contribute to a more abundant, affordable, and accessible supply of draught power, fuel, clothing, bedding, and other animal-derived necessities for life [82,87]; all of which in turn can impact the health and well-being of the children living within those households and communities.

### 5.3. Supporting Environmental Sustainability with a Focus on Carbon and Climate

Climate change is predicted to worsen child health and well-being for both present and future generations [88]. The continued rise in GHG emissions will likely lead to an increase in natural disasters, infectious disease, food and water insecurity, mass migration, psychological stress, and respiratory disease, among other harms [89,90]. While all food production systems produce GHGs, the amount can vary considerably based on factors such as the production methods being used, the geographical location of production, the time of the year of production, and the type of distribution networks and end markets being used [91,92,93]. In the case of livestock production there are even more granular factors that can make a difference such as the species, breed, age, and diet of the animal. As a result, there may be up to a 50-fold difference in associated GHGs between similar food products [94].

Cow’s milk provides an interesting case study in how agricultural practices can impact GHG emissions. The emission intensity for milk, which roughly equates to the amount of GHGs produced per unit of milk, is lowest in developed regions with large-scale milk production systems such as the U.S., ranging on average between 1.3 to 1.4 kg CO_2_ eq. per kg fat-and-protein corrected milk, while developing dairy regions with smaller scale farms have higher emission intensities ranging on average between 4.1 to 6.7 kg CO_2_ eq. per kg fat-and-protein corrected milk [95]. The differences in these numbers are largely dependent on the farming practices used, with more intensive production methods resulting in lower GHG emissions [96,97]. Importantly, emission intensities for milk across all regions has declined by almost 11% from 2005 to 2015, reflecting improvements in on-farm efficiency through improved animal productivity and better management [95]. These declines in emission intensities will likely continue into the future as further improvements are made to animal feeds, genetics, and manure management systems [98].

While GHGs get the most attention in sustainability discussions, it is important to note that a food’s carbon footprint is only one sustainability measure out of many that matter. Although often underappreciated or ignored in the media, livestock production does play several vitally important roles in healthy and sustainable food systems. For one, ruminants utilize land for grazing that is not suitable for growing crops, and they also upcycle low-nutritional quality foods (including agricultural byproducts), that would otherwise rot or be burned and release GHGs. In this manner, ruminants are able to convert low-quality/inedible plants and byproducts into nutrient-rich foods for human consumption while at the same time minimizing the release of GHGs from unused byproducts [99,100]. Livestock can also improve the health and value of non-arable lands through ecosystem services such as manure fertilization, land aeration, improved biodiversity, improved ecosystem water productivity, and improved carbon sequestration [65].

There are still notable opportunities in all food sectors, including the dairy sector, to reduce environmental impacts. In contrast to many industries, the dairy sector has continuously invested in research and innovation over the last several decades to reduce its impacts and natural resource use by making significant advancements in areas such as crop production, water use, animal genetics, animal care, and food safety [101]. The dairy sector has further committed to advancing sustainable dairy production around the world through the development of a Dairy Sustainability Framework focused on tracking and improving key impacts related to GHGs, soil, water, waste, biodiversity, animal care, working conditions, and product safety [102]. The Innovation Center for U.S. Dairy’s recently established ‘Net Zero Initiative’ aims to drive the industry to achieve carbon neutrality, optimized water usage, and improved water quality by 2050 [19]. Additionally, its Dairy Stewardship Commitment defines a rigorous set of standards that demonstrate positive social, economic, and environmental impact [101].

Global thought leaders increasingly recognize that dairy production and other livestock farming practices can and are on the path to being more sustainable [65,103]. Additional efforts, including research and advancements in innovation and technology, need to be made to ensure all food production systems, including those in LMICs, become more sustainable [65]. Mitigating climate change, preserving biodiversity, and preserving natural resources through sustainable farming practices will support the healthy environments needed for today’s children and future generations to flourish.

## 6. Conclusions

Hunger and malnutrition continue to persist among children at devastatingly high levels. Despite global efforts, significantly greater support for child health and welfare is urgently required for achieving the SDGs. Scalable solutions are needed to address these issues and their underlying causes, such as poverty, poor sanitation, and food insecurity. These solutions should come from collective efforts by major food systems actors (i.e., academia, industry, governance, civil society), and aim to consider the potential trade-offs and synergies of food system transformation. But the science will only take us so far. These innovations and interventions will need to be designed and delivered with care, going beyond food system stakeholders to the socioeconomic support systems for children–namely caregivers, families, schools, and communities. Food Systems transformation must engage children and adolescents as agents of change, not just as dependents and consumers. While it is critical to provide adequate nutrition for growth and development, engaging children in nutrition education and sustainability actions also can help pave a path to a healthier and more sustainable future for all.

Due to their interconnectedness and everchanging nature, it may not be possible to simultaneously maximize the health and sustainability of complex food systems. However, it is imperative that we make significant and continuous improvements towards both. Although dairy production can be resource intensive and is associated with greater environmental impacts than many plant-source options, the dairy industry as a whole continues to transform towards more environmentally sustainable practices, while at the same time maintaining positive contributions to local and global economies, human health (especially child nutrition), and community well-being. Finding the right balance between these types of trade-offs among health, society, economics, and the environment will play a key role in determining the successes and failures of food systems transformation and, in time, the future well-being of our population and planet.

## Data Availability

Not applicable.
